# Into the multi-omics era: Progress of T cells profiling in the context of solid organ transplantation

**DOI:** 10.3389/fimmu.2023.1058296

**Published:** 2023-01-31

**Authors:** Yao Zhi, Mingqian Li, Guoyue Lv

**Affiliations:** Department of Hepatobiliary and Pancreatic Surgery, The First Hospital of Jilin University, Changchun, China

**Keywords:** T cells, Single Cell RNA sequencing, TCR repertoire, allograft rejection, solid organ transplantation, allograft survival, alloantigens

## Abstract

T cells are the common type of lymphocyte to mediate allograft rejection, remaining long-term allograft survival impeditive. However, the heterogeneity of T cells, in terms of differentiation and activation status, the effector function, and highly diverse T cell receptors (TCRs) have thus precluded us from tracking these T cells and thereby comprehending their fate in recipients due to the limitations of traditional detection approaches. Recently, with the widespread development of single-cell techniques, the identification and characterization of T cells have been performed at single-cell resolution, which has contributed to a deeper comprehension of T cell heterogeneity by relevant detections in a single cell – such as gene expression, DNA methylation, chromatin accessibility, surface proteins, and TCR. Although these approaches can provide valuable insights into an individual cell independently, a comprehensive understanding can be obtained when applied joint analysis. Multi-omics techniques have been implemented in characterizing T cells in health and disease, including transplantation. This review focuses on the thesis, challenges, and advances in these technologies and highlights their application to the study of alloreactive T cells to improve the understanding of T cell heterogeneity in solid organ transplantation.

## Introduction

Transplantation is the most effective treatment for various types of end-stage organ failure (1–5). The primary barrier to successful transplantation is rejection. Alloreactive T cells are key mediators of allograft rejection (6–10). T cells possess high heterogeneity, which leads to distinct function and migration features, making it difficult to further dissect T cells’ cellular and molecular features in depth.

The heterogeneity of T cells stems from multiple molecular layers including DNA, RNA, and protein. Thus, the heterogeneity can be interpreted with epigenomic, transcriptomic, and proteomic data. And highly polymorphic TCRs represent a diverse antigen specificity of the T cell repertoire. Previous studies devoted to investigating these heterogenous T cells through traditional assays like flow cytometry but had limited success. Recently, with the advance of single-cell RNA sequencing (scRNA-seq), it is easy to measure the genes’ expression levels of each cell and unbiasedly characterize T cell types at the transcriptional level (11, 12). Based on transcriptome analyses, multi-omics methods have been extended from single-cell techniques, such as the combination of the transcriptome with epigenome, paring transcriptome with the proteome, and linking transcriptome with TCR. Besides, the advanced bioinformatics analysis algorithm for multi-omics datasets (13) enables the profiling of T cells’ heterogeneity from integrative data (14–18). Therefore, this review focuses on the high heterogeneity and diverse TCR of T cells and summarizes the advances of omics techniques in solid organ transplantation (SOT), and their combined application to dissect the heterogeneity of T cells. Furthermore, it discusses the promising future of multi-omics integration analysis and computational tools for complex omics data integration and analysis.

## T cells are heterogeneous

T cells possess phenotypic and functional heterogeneity. T cells expressing distinct surface markers indicate distinct subtypes and functions. The CD45RA, CD45RO, CD28, CD27, CD95 along with the homing and adhesion molecules CD62L, CCR7, CD69, CD103, CXCR5, CXCR3, CCR4, CCR6 expressions are well-established markers for T cell distinction. For example, CD45RA^+^CCR7^+^ cells, referred to naive T cells, representing not encounter any antigens. CD45RA^+^CCR7^+^CD27^+^CD95^+^ cells were termed T_SCM_, they have the potential to reconstitute the memory and effector subtypes as well as sustain longevity through self-renewal (19). The main function of central memory T (T_CM_) cells with a signature of CD45RA^-^CCR7^+^ is proliferation instead of the effector, they exhibit lymphoid homing profiles. CCR7^-^ cells represent effector memory T (T_EM_) cells, exhibiting rapid effector functions. Tissue-resident memory T (T_RM_) cells express CD69, CD103, or CXCR6, parking within tissue instead of circulating. CD4^+^CXCR5^+^ T cells are termed T follicular helper (T_fh_) cells, which are adept at providing help to the differentiation of B cells. The Th1 cells (CD4^+^CXCR3^+^) are considered the main driver of acute rejection. Th1 secrets IL-2 and IFN-γ to activate the CD8 cytotoxic T cell or B cells (20), or directly damage the graft through the Fas-FasL pathway (21). The Th1 and Th2 (CD4^+^CCR4^+^) are antagonistic, the cytokine IL-4 and IL-10 produced by the Th2 inhibit the Th1 differentiation. Enhance, Th2 cell has long been thought to have a prevention role on rejection, but recent studies evident a promotion role of the cell in the rejection (22–24). A large body of evidence demonstrates the Th17 cells (CD4^+^CCR4^+^CCR6^+^CXCR3^-^) are important in transplant rejection by secreting the IL-1 7 to recruit the neutrophils to cause graft damage (25–27). Intracellular genetic markers can also be utilized to dissect T cells’ heterogeneity and function. For transcriptome, resting cells have a signature of *SELL, TCF7*, and *CCR7*, which corresponds to T_CM_ or naive T cells. The activated cells express cytotoxicity-associated genes, such as *GZMB, GZMK*, and *CCL5*, referred to as T_EM_ or effector T cells (T_eff)_. T_RM_ cells express adhesion molecules like *CXCR6* and *ITGA*, contributing to residence (28). RNA detections reveal the T cells’ current state, but epigenetic features demonstrate the T cells’ progenitor and potential to drive the expression of specific genes (29).

**Figure 1:**
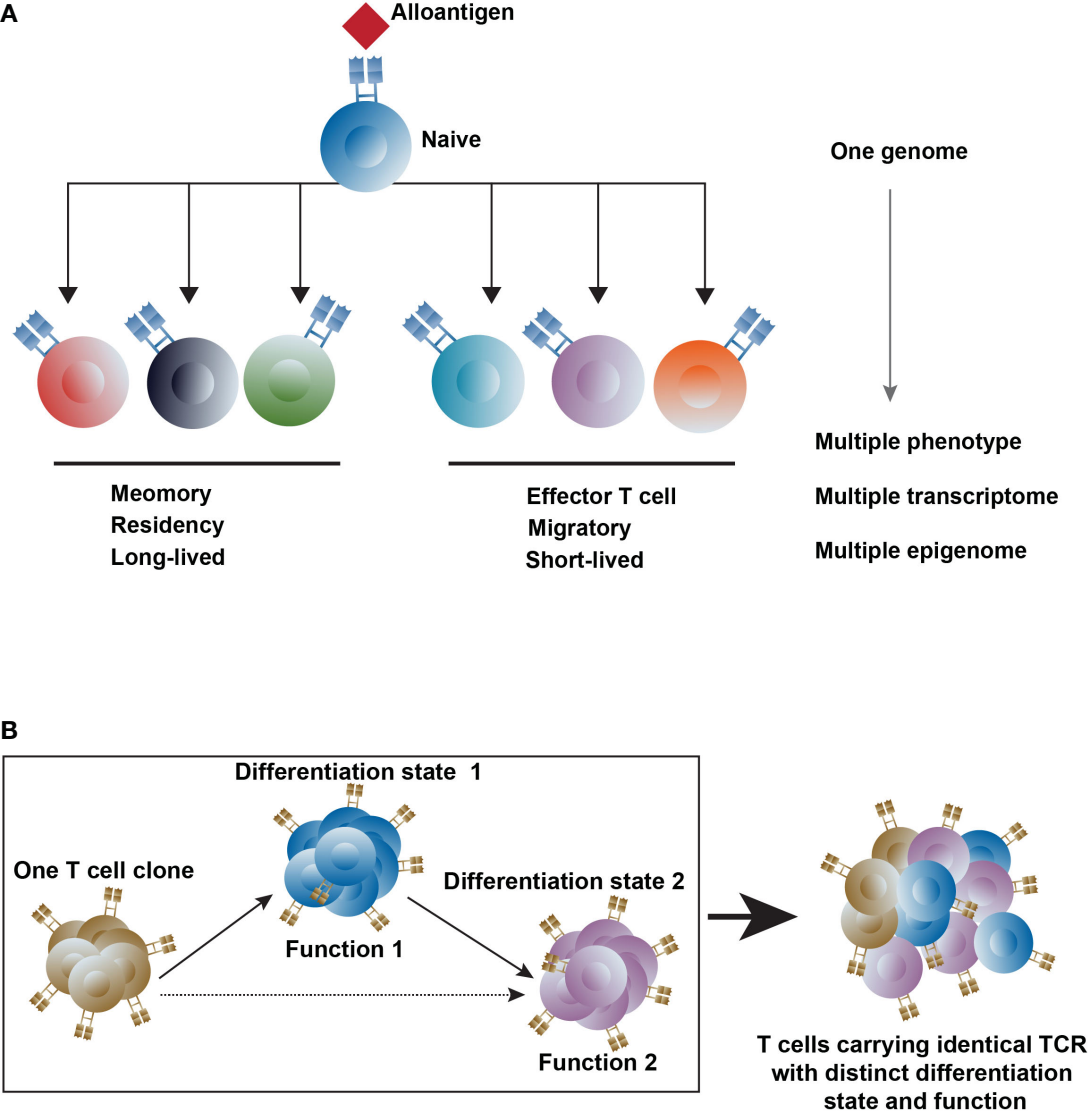
The heterogeneity of T cells. **(A)** T-cell differentiation states are regulated by epigenetics upon interacting with external factors **(B)** Trace the T cell clones with different differentiation states and functions after transplantation. Differentiation state1, 2 represents different differentiation states, and Function1, 2 represents T cells with a different function. The dashed line represents the potential differentiation pathway.

At the initial stage of activation, naive T cells possess plasticity, which means the capacity to produce different phenotypes initiated from an individual genome (30). In alloresponse, the plasticity of a naive T cell, mainly regulated by epigenetics, refers to the ability to differentiate to other T cell subsets in response to the alloantigen. This means naive T cells present unique chromatin landscape and gene expression patterns before alloantigen recognition, which can alter with activation. Upon interacting with external factors like dose and properties of alloantigen, effects of immunosuppressive regimens, and local microenvironment, activated naive T cells differentiate into multiple lineages and display distinct properties of longevity, proliferative ability, properties of tissue residency or migratory (31, 32) (**Figure 1A**). In the setting of alloresponse, a naive T cell activated by an alloantigen would differentiate into short-lived effectors and long-term survival of memory cells. The memory cells are located in the lymph node (T_CM_), in the peripheral (T_EM_), and in the peripheral non-lymphoid tissues (T_RM_). In kidney and liver transplantation, the memory T cell proportion increased in the blood, graft, and lymph node of the rejected recipient (33, 34). And the donor-reactive T_RM_ clones dominated in the transplanted intestine correlated with graft rejection, and they re-express high levels of CD28 upon rejection (35), implying that a likely intermediate state between T_RM_ and T_eff_ cells is associated with rejection. A naive CD4 T cell differentiates into many lineages of T helper cells upon the TCR stimulation controlled by the respective transcription factors under unique cytokine-polarized milieus: IL-12 and IFN-γ promote the Th1 differentiation with the activation of the master regulator transcription factor T-bet through STAT4; the IL-4 and IL-33 promote the activation of STAT6 and GATA3, which induce Th2 cell differentiation; TGF-β and proinflammatory cytokines IL-6 and IL-23 drive the differentiation of Th17 cells through the activation of STAT3 and RORγt; TGF-β promotes the induction of Tregs, which are controlled by the transcription factor Foxp3 (20, 36, 37). The plasticity of the CD4 helper cell usually refers to the fate alterations between the Th1 and Th2, and the Th17 and Treg, which stems from the epigenetic modification of histones and DNA regulated by the lineage-restricted transcription factors (38).

Another ingredient of heterogeneity within T cells is TCR diversity, which is yielded in different ways, including somatic rearrangements of V, (D), and J gene segments, random addition or deletion of nucleotides, and pairing of α and β TCR chains (39). In αβ T cells, both TCR α and TCR β chain contain a hypervariable complementary determining region 3 (CDR3) formed by somatic recombination and nucleotide insertions, leading to highly diverse TCR. Approximately 2 × 10^19^ unique TCR pairs can be generated theoretically (40–43), while only 2 × 10^11^ T cells exist in an individual because of the positive and negative selection processes (44–46). A large repertoire of T cells with diverse antigen-specificity in organisms allows the immune defense to deal with pathogen infection through non-self-antigen recognition (47, 48). When stimulated by antigens, each T cell clone can expand into multiple progeny cells carrying the identical TCR but may with distinct differentiation states and functions (49, 50). Alloreactive T cells comprise about 10% of the blood-circulating T cell pool in healthy adults (51, 52). After transplantation, the recognition of alloantigen leads to the activation and clonal expansion of alloreactive T cells. Thus, the TCR is a meaningful proxy for tracking the T cells in alloresponse, and profiling the heterogeneity of T cell-mediated alloresponse (53–58) (**Figure 1B**).

## Insights into T cell heterogeneity with single-cell approaches in SOT

### Characterize the T cell heterogeneity in SOT at the transcriptome level by scRNA-seq

T cells coordinate with each other exhibiting a complex effect in graft rejection. Alloantigen-specific T cells exhibited different effector mechanisms, including direct cytotoxicity to the allograft and indirect recruitment of graft-damaging inflammatory cells, and production of inflammatory cytokine (59). Resolving the complex process involving T cells with heterogeneity is not possible with the traditional bulk-seq approach, but the recently emerged scRNA-seq technique might be even better (60, 61). By profiling the cell with scRNA-seq technology, specific T cell clusters or genes related to the rejection in liver, kidney, lung, and intestinal transplantation have been reported (62, 63).

Heterogenous T cells with different functions in graft can be dissected by scRNA-seq. In chronic kidney transplantation, Zhao et al. (60). used scRNA-seq to study T cells from biopsy samples and four clusters were identified, including CD4^+^ T cells, CD8^+^ T cells, cytotoxic T lymphocytes (CTLs), and regulatory T cells (Tregs). Furthermore, they demonstrated that CD8^+^ T cells and cytotoxic T lymphocytes (CTLs), usually as a signature of immune activation, were more enriched in the chronic kidney transplant rejection biopsy samples. By ssGSEA analysis of the single cell transcriptomes, they revealed CD8^+^ and CTLs exhibited higher cytotoxic activities by enhanced interferon (IFN) secreting, antigen presentation, and producing cytokines and chemokine, while IFN was downregulated in Tregs. In this study, the memory T cell was not characterized separately from other T cells, and the cell origin from the donor or recipient was undefined.

Single-cell transcriptome profiling enables determining the function of T cells from the recipient and donor accurately. It is common knowledge that multilineage blood chimerism often develops and hematopoietic chimerism can serve as an approach to achieve immunological tolerance across HLA barriers in patients after transplantation (64–66). Previously, due to a lack of accessible approaches to distinguish recipient and donor cells at the individual level, the persistence of chimerism within recipients after transplantation and its role in allograft were investigated in limited depth. In kidney transplantation Malone et al. (67). accurately determined the cell origin based on expressed single nucleotide polymorphisms sequenced by whole exome sequence from the biopsy sample derived from the recipient and donor. Based on these results, they furtherly described the results of scRNA-seq analysis and revealed that donor T cells are predominantly quiescent, determined by a high correlation between donor-origin T cells in rejecting biopsies and non-rejecting T cell transcripts. Conversely, the T cell of recipient origin takes an effector role, especially those with acute cellular rejection.

A human lung T_RM_ generation study employed scRNA-seq to analyze serial airway samples obtained longitudinally from human leukocyte antigen (HLA)–disparate lung transplant recipients. They distinguish the origin of T cells from donor and recipient, and found the donor-origin T_RM_ replaced by recipient circulating T cell in lung-graft, the bronchoalveolar lavage T cells from the transplant lung revealed three different subsets: A) a donor mature T_RM_ subset expressing CD69 and CD103 expressed high levels of T_RM_ differentiation genes including *ITGA1, CXCR6, ZNF683*, and *RUNX3*, these cells exhibited effector function *via* expressing *GZMA, NKG7, CCL5, KLRD1, PFN1, CD27*, and *IL32*; B) a T_RM_-like subset comprising of mixed cells originating from donor and recipient retained expression of T_RM_ signatures, the subpopulation was fewer in number compared with T_RM_ subset, and expressed differentiation-associated genes *SOX11* and *CDH6* (68, 69); C) a non-T_RM_ subpopulation absent of CD69 and CD103 expression constituted by recipient T cells reduced the expression of tissue resident and effector-associated genes, and increased the expression of regulation-associated with genes such as *RPL13, PABPC1* and *MLLT3*, cell cycle (*BTG1*), and cytokine signaling (*IL7R* and *JAK3*) (70). It suggested that T_RM_ in the lung is heterogeneous in phenotype, circulating ability, and effector function. Analysis of the longitudinal samples suggested that the T_RM_ pool in the graft was supplied by the recipient circulating T cells over months, while the persistence of donor T_RM_ is associated with fewer primary graft dysfunction and acute cellular rejection. But the exact role of T_RM_ in alloresponse is unrevealed in this study.

As an important part of the memory T cell populations in organs, T_RM_ participation in the alloresponse was elegantly described in intestine transplantation. Zuber et al. demonstrated a slow replacement of donor-derived graft-versus-host (GVH) T_RM_ by recipient host-versus-graft (HVG) T cell correlated with the absence of rejection and the long-term presence of macrochimerism in the recipient’s blood by bulk RNA-seq (35). The result proposed that a balance between GVH and HVG reactivities is associated with tolerance induction, but the GVH T_RM_ function in the graft was unable to be revealed by the method simply comparing the frequency of both clones. Later, by using scRNAseq, Fu et al. found the GVH T cell clones, originating from T_RM_ in the transplanted intestine, displayed cytotoxic T_eff_ transcriptional profile in the recipient’s bone marrow, indicating they mediated lymphohematopoietic GVH responses to promoted engraftment of graft-derived hematopoietic stem progenitor cells that maintain macrochimerism to facilitate tolerance (71).

### Epigenome sequencing is a potential tool for interpreting T cells in SOT

T cells can acquire specialized functions after interacting with alloantigens despite emerging from the same genetic background, which is believed to be driven by epigenetic alterations. Thus, interpreting the epigenome specific to alloreactive T cells is crucial to comprehend the activation and differentiation of these T cells in SOT. The epigenome regulates gene transcription mainly by DNA methylation and modification of chromatin status.

DNA methylation, involved in establishing and sustaining chromatin structure and regulating gene transcription, is a covalent alteration of the DNA molecule which is stable and heritable. Many studies have emphasized the significance of DNA methylation in regulating intricate gene expression patterns in immune response (72, 73). Two methods have been achieved to explore intercellular heterogeneity of DNA methylation at single-cell resolution. One is single-cell bisulfite sequencing (scBS-seq) (74) which detects genome-wide DNA methylation. Another is single-cell reduced representation bisulfite sequencing (scRRBS-seq) (75) which enriches sites containing high CpG content. Based on the genome and transcriptome sequencing (G&T-seq) approach (76) allowing for physical isolation of DNA and RNA from single-cell lysates, Single-cell methylome and transcriptome sequencing (scM&T-seq) (77) enables joint analysis of the intricate relationship between DNA methylation and transcription in heterogenous cell subtypes. Recently, a tumor study observed that DNA methylation participates in shaping tumor-reactive and bystander CD8^+^ tumor-infiltrating lymphocytes which refers to a subpopulation of T cells recognizing and destroying tumor cells specifically and recognizing a wide range of epitopes unrelated to the tumor, respectively (78).

**Figure 2:**
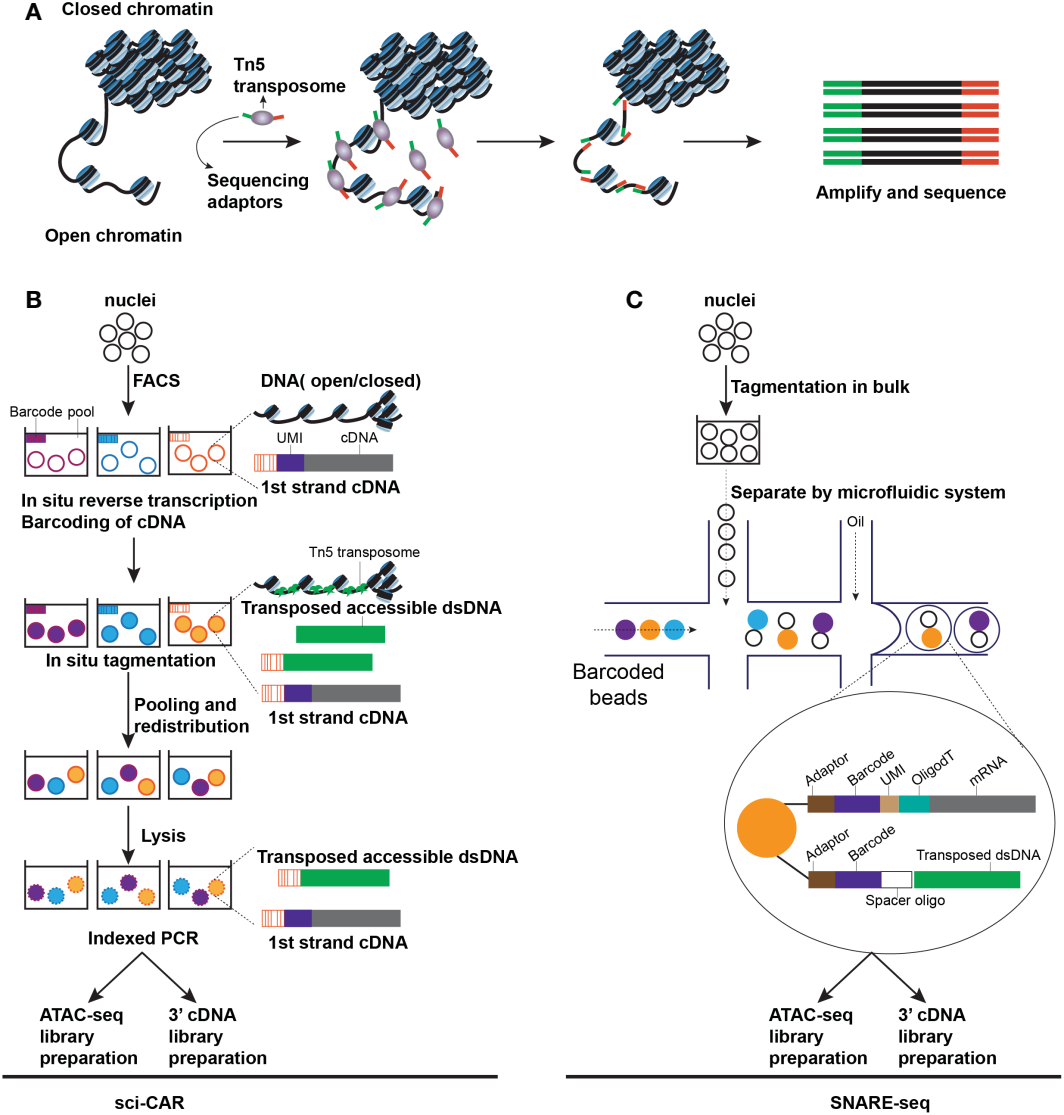
Techniques for capturing chromatin accessibility from an individual cell. **(A)** The schematic of ATAC-seq. Open chromatin regions can be inserted by transposome and generate fragments that can be amplified by PCR **(B)** The schematic of sci-CAR seq. All nuclei are extracted and distributed by Fluorescence-activated Cell Sorting (FACS) to each pool. A first cDNA sequence can be introduced by reverse transcription with a pool-specific Barcode and a unique molecular identifier (UMI). After barcoding cDNA, a first transposed dsDNA sequence is introduced by Tn5 transposase *in situ* tagmentation assay, bearing a pool-specific barcode. Then redistributed nuclei, each of them is lysed, and the lysate is split into two parts, one half prepares for the library of 3’ cDNA, another half prepares for the library of ATAC-seq **(C)**. The schematic of SNARE-seq. A microfluidic system has been applied in SNARE-seq and enabled parallel capture of transcriptome and chromatin accessibility from a single cell.

For the assessment of chromatin status, assays for transposase-accessible chromatin sequencing (ATAC-seq) could be used to measure the genomic sequences’ accessibility, which represents particular genes’ expression or sequences’ openness, such as the binding regions for transcription factors or enhancers, and is considered a hallmark of genomic activity. In the ATAC-seq, the open chromatin region can be labeled with sequencing adaptors *via* the Tn5 transposase, amplified *via* PCR, and then sequenced (79–81) (**Figure 2A**). Single-cell ATAC-seq (scATAC-seq) can be performed on several single-cell platforms, such as C1 and Chromium systems. The C1 platform is based on the microfluidic plate system and thus all library preparation steps including cell lysis and PCR amplification is automatic (80). However, the Chromium system is based on the microfluidic droplet, in which the isolation of nuclei and the tagmentation of Tn5 must be prepared manually before separation in droplets. ATAC-seq has higher throughput compatibility than other DNA methylation measurement approaches. Generally, high-throughput ATAC-seq approaches are dependent on the label of accessible chromatin when preparing nuclei in bulk before linking the labeled DNA and RNA with identical barcodes from the same cell, either by droplet-based or combinatorial indexing techniques which is a method to increase throughput by serial barcoding pools loaded with cells. For instance, single-cell combinatorial indexing-chromatin accessibility and RNA sequencing (sci-CAR) (**Figure 2B**) (82) used combinatorial indexing to measure > 11000 nuclei in each test. Lower throughput approaches, processing complete cells instead of nuclei, have been illustrated, such as scCAT-seq (83) and ASTAR-seq (84)). They have the potential to be more feasible for assays in which scarce cells are to be sequenced compared to high throughput assays.

Paired sequencing (Paired-seq) has promoted throughput by adopting a combinatorial indexing protocol based on ligation, which measures one million nuclei in each test. Based on paired-seq, the combinatorial indexing technique sensitivity was enhanced considerably with split-pool ligation-based transcriptome sequencing (SPLiT-seq) (85) and simultaneous high-throughput ATAC and RNA expression with sequencing (SHARE-seq) (86), designed to detect the chromatin potential from a single cell and investigate the predictive effect of chromatin accessibility on mRNA expression levels and lineage determination in a cell. Based on the microfluidic drop-sequencing technique, single-nucleus chromatin accessibility and mRNA-expression sequencing (SNARE-seq) can execute the parallel measurement of chromatin accessibility and gene expression from identical nuclei (**Figure 2C**) (87). 10X Genomics Chromium platform has adopted this technique using hydrogel beads carrying divided oligonucleotides capturing the labeled genome and mRNA. Recently, based on the sequencing HEteRO RNA-DNA-hYbrid (SHERRY) (88) technique and a similar technique, *in situ* sequencing hetero RNA-DNA-hybrid after assay for transposase-accessible chromatin-sequencing (ISSAAC-seq) (89), as an optional approach for multi-omics sequencing of a single nucleus, tags the accessible chromatin at the first round followed by reverse transcription and tags DNA-RNA hybrids at the second round. For this approach, single nuclei are separated by microfluidic apparatus, and DNA and RNA libraries are built separately through the difference between two-step adaptor configurations.

The αβ TCR interacting with intrathymic MHC determines the fate of double positive (DP) thymocytes which express both CD4 and CD8 molecules (90, 91). DP cells moderately affinitive for self-MHC peptides can survive positive selection and differentiate into CD4^+^ and CD8^+^ single positive (SP) cells (92, 93). On the contrary, DP cells highly affinitive for intrathymic ligands die of negative selection or part of them become “agonist-selected” cells, such as regulatory T cells (Treg) or precursors of CD8α^+^CD8β^–^(CD8αα) gut intra-epithelial lymphocytes (94–96). These heterogenous thymocyte populations are poorly characterized, in part because of an incomplete understanding of underlying differentiation programs. Combining scATAC-seq with scRNA-seq enables analyzing T cell transcriptional heterogeneity from the perspective of differentiation and development. Compared with traditional RNA-seq analysis (97, 98), single-cell analysis of transcriptional and chromatin accessibility delineates trajectories with minimal bias. Recently, a study focused on human and mouse αβ T cells in the thymus by examining their transcriptional expression and chromatin accessibility at the single-cell level. It depicted the transcriptomic and epigenomic landscape of αβ thymocytes in mouse and human thymus and delineated developmental trajectories of CD4^+^, CD8^+^ lineage, and “agonist-selected” thymocytes, which interprets the heterogeneity of thymocytes by integrating scRNA-seq and scATAC-seq (99).

In SOT, how epigenetics regulates the differentiation of alloreactive T cells remains unclear. A complete understanding of the development trajectory helps interpret T cells’ history and potential to express the specific gene. Thus, combining scATAC-seq with scRNA-seq is a potential way to help decode the heterogeneity of development and function dynamics of the alloreactive T cells after transplantation.

### Proteomic sequencing as an auxiliary tool for T cell characterization in SOT

Determining the phenotype profile of T cells by measuring protein expression replenishes the scRNA-seq technique and defines T cell subpopulations more specifically because certain T cell type signatures are hardly measured with the transcriptome method. For example, solely with scRNA-seq, it is difficult to detect the surface proteins without an RNA analog (41), like CD45RO and CD45RA (the PTPRC gene’s isoforms) that distinguish T cell subsets with naive and memory T cells. It also provides little insight into markers that has high dropout at the RNA level by scRNA-seq alone, such as CD4 (100). Identifying CD4 T cells and distinguishing memory cells from naive cells by CD45RA and CD45RO are critical steps in cell subset determination. Thus, combining scRNA-seq with protein expression compensates for the transcriptomic shortcoming in immune cell phenotyping.

**Figure 3:**
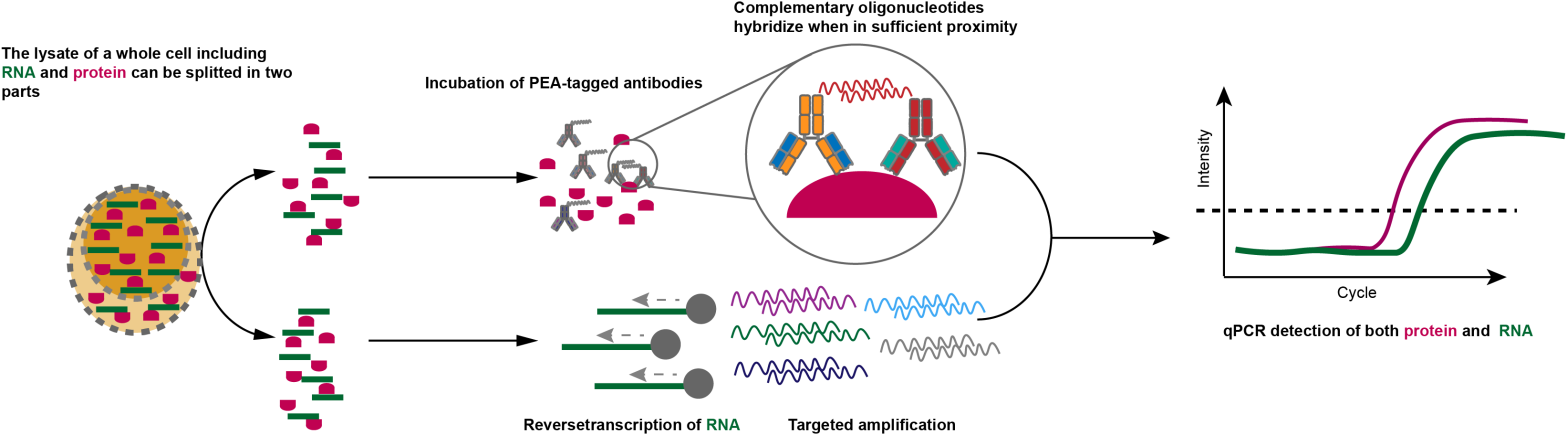
PEA approaches for transcriptome and proteome at single-cell resolution. The whole-cell lysis including RNA and protein can be separated into two parts: one is incubated with PEA antibodies for the measurement of specific proteins *via* qPCR and one is for quantification of cDNA by qPCR.

One approach termed proximity extension assay (PEA) (101) measures protein expression through antibodies tagged with oligonucleotides, which hybridize when in sufficient proximity. And unique sequence can be generated and can be further amplified and measured by qPCR. Hence, the measurement of protein in an individual cell is converted into the detection of nucleotide signals. In this approach, cell lysates were divided into two parts, one half is utilized to detect transcripts of interest by qPCR, and the other half is used to perform PEA (**Figure 3**). Furthermore, PEA is compatible with the current scRNA-seq platform and thus can be applied to produce proteomic and transcriptomic data even though the throughput is low. Proximity ligation assays (PLA) adopt a similar approach but are dependent on the ligation in which two antibody-conjugated oligonucleotides get into proximity on the same protein target, instead of hybridization. PLA enables simultaneous measurement of a single protein and corresponding transcript on a droplet digital PCR platform (102–104). The throughput of PLA has been increased by proximity ligation assay for RNA (PLAYR) by measuring transcripts and proteins using mass cytometry, capacitating the detection of > 40 distinct protein epitopes and transcripts from many cells simultaneously (105). Recently, a method named single-cell protein and RNA co-profiling (SPARC) can physically separate mRNA and protein solute (106), capacitating the parallel measurement of whole transcriptome and extracellular and intracellular proteins through PEA (107). The combination of oligonucleotides-conjugated antibodies with microfluidic platforms and micro-well systems, such as 10X Genomics and BD Rhapsody respectively, dramatically increased the throughput. Based on this approach, RNA expression and protein sequencing (REAP-seq) (100) and cellular indexing of transcriptomes and epitopes by sequencing (CITE-seq) (108) have emerged, in which cells are bonded with antibodies panel, each tagged with different barcode oligos enabling being simultaneously captured with mRNA from a single cell after lysis.

The limitation of antibody-based approaches is the feasibility of reagents specific to the antigen. Consequently, the number of detectable epitopes is extremely decreased as many antibodies specific to the antigen are required for its measurement. Thus, it is imperative to develop an antibody-independent approach to cover the cellular proteome extensively. Such an approach includes single-cell proteomics by mass spectrometry (SCoPE-MS) (109) and SCoPE2 (110), which can analyze several proteins and modify post-translation in an individual cell. However, they have not been integrated into the multi-omics technique. Most recently, antibodies used in those approaches discussed above have been replaced by the nanobody phage-display libraries in an optional method termed PHAGE-ATAC assay (111). It may provide a promising approach for measuring protein without needing antibodies.

## Decoding allogeneic T cell response with single-cell TCR-seq in SOT

### Profiling the allogeneic T cell response by TCR-seq

By implementing high-throughput TCR sequencing (TCR-seq) techniques, the clonal frequencies and the diversity of alloresponse T cells’ repertoire were characterized and the alloresponse T cells’ repertoire is highly specific for a donor-recipient pair (51), and both the naïve and memory T cell clones lead to alloreactivity and can be detected *in vitro* (112, 113). Some pathogen-specific memory T cells possess the allogeneic function in a cross-react manner (114). In SOT cases, owing to the private property of the alloreactive clone repertoire in each donor-recipient pair, mixed lymphocyte reaction (MLR) assay, served as an approach to study donor-reactive T cells, giving an accessible way to identify and purify proliferating against alloantigen T cells labeled by CFSE, which can be sequenced by TCR-seq technique to identify the alloreactive clones (115). Combining MLR assay with the TCRβ-seq technique has been adopted to estimate alloresponse in various types of organ transplantation (116), including kidney transplantation (117), liver transplantation (118, 119), and intestinal transplantation (35, 71). The TCR-seq application in SOT has been recently reviewed (120).

Through TCR repertoire overlap analysis between pre-transplantation donor lymphoid tissue and post-transplantation peripheral blood monocular cells (PBMCs), circulating naive donor T cells derived from progenitors presented in the allograft could be proved to develop in the recipient thymus. Naive T cells’ repertoire is highly diverse, hindering the assessment of clonal overlap among various tissues within the same recipient (121); however, it is accessible to detect the clonal overlap of memory T cells. Based on this theory, in recipients receiving intestinal transplantation, Fu et al. (122) detected high enrichment of recent thymic emigrant (RTE) phenotypes and T cell receptor excision circles (TRECs) in donor-derived circulating CD45RA^+^CCR7^+^ T cells, which represents *de novo* generation of donor-derived T cells. Subsequently, they sorted donors’ naive and memory T-cells by Fluorescence-activated Cell Sorting (FACS) from the recipient following transplantation and donor lymphoid tissue from pre-transplantation and performed a high throughput CDR3 sequencing for these donor-derived T cells. They compared the TCR repertoire overlap of naive and memory T cells between pre- and post-transplantation and found post-transplantation T cell clones overlapping with those in pre-transplantation only among memory T cells. Thus, combing the lack of repertoire overlap with pre-transplantation naive T cells with the abundance of naive T cells’ RTEs and TRECs suggests that donor naive T cells originate from precursor cells in the allograft that develops *de novo* in the recipient after transplantation.

TCR repertoire analysis between the intragraft and the periphery reflects T cell migration and local expansion status. Intragraft and circulating T cell clonotypes differ substantially (123–125). In a liver graft and blood sample TCR comparison study, Elmar and his colleagues demonstrated that the TCR repertoires between the graft and the peripheral blood are different in non-rejected patients, while the correlation between rejected graft and blood was higher (126). A similar result was reported in cardiac transplantation, that a high degree of TCR repertoire overlap was found between cardiac allograft and the periphery in patients experiencing acute cellular graft rejection (112), these overlapped T cells were considered infiltrating T cells without apparent clonal expansion. Thus, the peripheral T cells massively infiltrating the graft cause rejection. As profiling, the entire peripheral blood TCR repertoire in patients with rejection or GVHD after liver transplant, the diversity and the N-addition length distribution of the CDR3 are associated with the diseases (118, 127, 128).

### Understanding allogeneic T-cell response with single-cell TCR-seq

Defining a clone solely by the beta chain sequence with TCR-seq limits the accuracy of a clone definition. A study on cellular rejection after cardiac transplantation showed that a subset of T cell clones shared TCR repertoire between blood and tissues containing several public (present in unrelated healthy donors) clones suggesting their bystander status, and the study also showed that the shared TCR repertoire constituted by a subset of cross-reactive sequences (129), offering reasonable evidence for intragraft bystander T cell local response. However, the study just has the TCR beta chain sequenced and limit the capacity to track authentic clonality, discover antigen and determine the true alloreactivity. Thus, confirmation of appropriate TCR alpha and beta chain pairs at the single-cell level enables the confirmation of aiming peptides, either allo- or viral-reactive, or both.

The adoption of TCR sequencing in the context of scRNA-seq facilitates tracking the function of the alloreactive T cell at a single clone level among the total T cell pool. Indeed, approaches, including those sponsored by 10X Genomics and BD Biosciences, generate data on authentic clonality *via* pairing scRNA-seq with single-cell TCR-seq (scTCR-seq). The challenges of employing these techniques in distinguishing alloreactive TCR clones are the diversity of antigens and epitopes of the donor-recipient pairs. Using MLR to establish an alloreactive TCR library to distinguish the alloreactive clones and describe their function was performed in intestinal transplantation as mentioned in the above section (35, 71).

Additionally, to pair scTCR-seq with ATAC-seq, the method used is transcript-indexed ATAC-seq (T-ATAC-seq) (130). It accurately identifies TCR ligands and is a complementary approach to integrating the T cell epigenomic state with the TCR sequence (131, 132). Furthermore, it isolated single cells by the microfluidic approach after tagmentation of genomic DNA with Tn5. Next, it reverses transcribed the TCR mRNAs by exploiting primers aiming at the C region and underwent multiplex PCR amplification by inner primers specific for C and V sites. Simultaneously, it fragmented the ATAC-seq experience 5’ extension and amplified them through PCR. Furthermore, the T-ATAC-seq technique is capacitated to estimate the specificity mechanism and expanded T cells regulated by epigenetic elements, such as cis- and trans-acting factors. This joint analysis contributes to the discovery of alloantigens that drive T cell differentiation, or cis- and trans- regulators which regulate the expansion of a T cell clone. For instance, the epigenetic features of clonal T cells in malignant lymphoma were researched by this approach and it revealed that the epigenetic features have a potential to distinguish malignant T cell clonal expansion from benign (47, 130). This combined approach has not been employed in the SOT field. While simultaneous measurement of single-cell TCR sequence and epigenetic signatures is useful to interpret the alloreactive T cells’ differentiation and expansion kinetics regulated by epigenetics.

### The prospective application of multi-omics in SOT

Multi-omics technologies at single-cell resolution were constructed to separate multiple molecules such as DNA, TCR, RNA, or protein in a single cell (133), and then sequence them in parallel. Because of the vast cellular heterogeneity of immune cells, a majority of multi-omics technologies with the single-cell resolution are supposed to be utilized to systematically analyze genome, transcriptome, proteome, epigenome, and ultimately, spatial transcriptome to measure the heterogeneity, thus offering more systematic and definite knowledge than mono-omics approaches (134). As an early example, ATAC with select antigen profiling by sequencing (ASAP-seq) (135) provides a method for performing ATAC- and CITE-seq in parallel on the microfluidic platforms. Additionally, a method referred to as DOGMA-seq was further expanded by incorporating RNA-seq measurements (135). A parallel method, TEA-seq (136), also has been demonstrated recently. From these studies, the data generated by different omics achieves high precision because they can validate mutually. Pairing different omics data, such as the data of scRNA-seq, scTCR-seq, and scATAC-seq can promote the interpretation of complicated regulatory mechanisms of diseases (137). Although few of these integration analyses have been used in the transplant immunology field, given the widespread use of these approaches in other realms such as cancer immunology, and biological development, multi-layers of omics will be a highly efficient tool in the study of organ transplantation.

The histologic information is missing in scRNA-seq. The emergence of spatial transcriptomics (ST) has made a breakthrough and it generates transcriptomic data on histological tissue sections. The greatest strength of the ST technique is that the spatial information of target cells can be acquired, such as T cells, and their cellular interactions can be investigated in their native location. Because scTNA-seq requires tissue to dissociate into a single cell suspension, limiting comprehension of cellular interactions, which is meaningful for T cells’ research. Combining ST with other omics, which matches the RNA profile of a cell with its spatial information within a tissue (138) is an optional way to make up for this shortcoming. Several approaches developed to integrate spatial heterogeneity with transcriptional heterogeneity in multicellular systems (139–142). In addition, the ST technique can match the transcriptomic data with its pathology report because it allows for the tissue slice stained by immunofluorescence or hematoxylin and eosin (H&E), which is before the determination of Banff criteria on the same slice. However, it has not been used in the transplant immunology field. Integrating ST approaches into multi-omics will locate the allogeneic T cells, and provide an intragraft immune landscape, to further allow a comprehensive understanding of the transcriptional and spatial regulation of T cells in the graft.

## Insights into the integration of multiple layers for single-cell datasets

**Figure 4:**
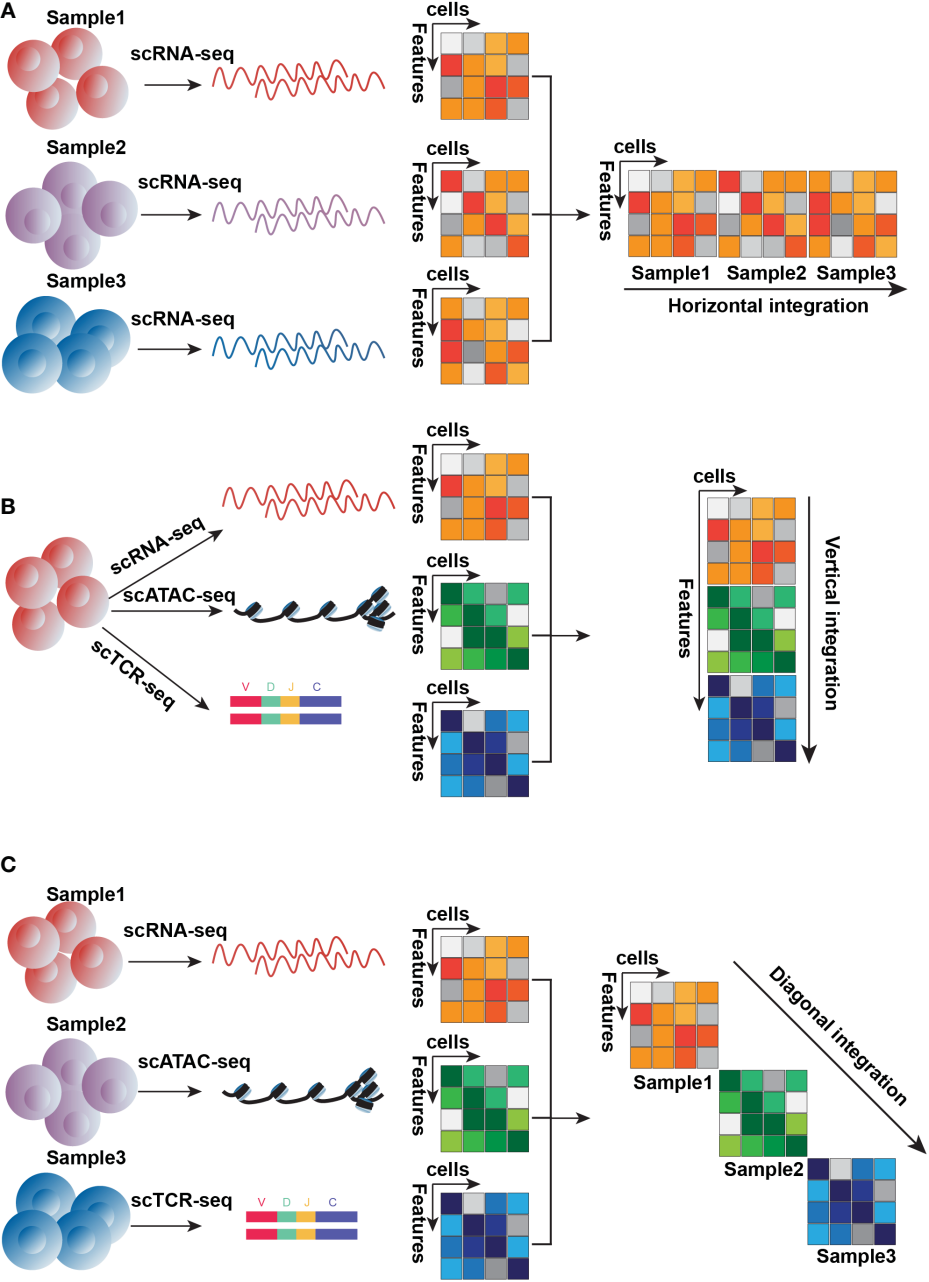
Three conceptual types of integration approaches. **(A)**. The horizontal integration approach is designed for distinct samples detected by the same technique, here is an example of scRNA-seq **(B)**. Vertical integration is suitable for subpopulations of the same sample detected by distinct techniques, here is an example of scRNA-seq, scATAC-seq, and scTCR-seq **(C)**. Diagonal integration is designed to integrate different samples measured by different techniques, here is an example of scRNA-seq, scATAC-seq, and scTCR-seq.

With the generation of various omics data, several methods have been developed to integrate these datasets. According to a recent review, these integration approaches can be grouped into three conceptual types: the first type can be termed “horizontal integration” (**Figure 4A**), integrating data generated from the same techniques across distinct samples. This integration method aims to remove technical noise, such as batch effects derived from different sample preparations, which ensures that the remaining variants originated from biology. For this step, many frequently-used methods, like Harmony (143), Scanorama (144), ComBat (145), LIGER (146), limma (147), scourge (148), and so on, have been developed and validated (149). The limitation in horizontal data integration is the difficulty to balance noise obliteration and biological signal retention, such as differential expression of the gene among cells. Because the extent of true biological signal from sample-to-sample variability and technical noise is hard to control. Too much removal of biological variation would result in a loss of information about cell type, but not enough removal of technical noise would result in a low biological signal-to-noise ratio.

The second type of integration approach is “vertical integration” (**Figure 4B**), which combines multi-omics data simultaneously profiled from an individual cell. This is exemplified by the abovementioned techniques such as scM&T-seq, CITE-seq, SNARE-seq, SHARE-seq, and scTCR-seq. Vertical data integration is to construct connections of different molecule layers and obtain knowledge from their relationship. For T cell research, the advantage of this integration method is to identify cell subtypes that might have one similar molecular layer but others different, such as cells that have similar transcriptional traits but distinct features from chromatin accessibility. For example, Buenrostro et al. used this integration strategy to analyze the alterations of transcriptomes and chromatin accessibility across stages of hematopoiesis, they identified a majority of cell subtypes based on transcriptomes but also epigenetic priming absent from the transcriptional level. Several methods originally developed for bulk data, can be applied for this integration, like canonical correlation analysis (CCA) (150), PLS (151), MF (152), MCIA (153), JIVE (154) and MOFA (152), which are based on the matrix factorization framework because of its concision, interpretability and low risk of overfitting.

The third type of integration method, “diagonal integration” (**Figure 4C**), is for joint analysis of multi-omics data generated by experiments where both cells and genomic traits are distinct. This integration is present in unmatched assays where distinct molecular layers are sequenced in distinct cell subtypes. Diagonal integration, from which biological views obtained have difficulty interpreting and validating, is more challenging compared to horizontal and vertical integration. Some methods have been developed to perform diagonal integration, such as MATCHER (155), MMD-MA (156), SCIM (157), and UnionCom (158)

## Conclusion

At present, multi-omics profiling approaches at single-cell resolution continue to emerge at a horrendous pace. It generates different omics data in parallel for thousands of single cells by the latest approach termed “Omni-seq”, in which omics detections can be paired with spatial information and lineage-based knowledge to identify the T cells’ molecular state, localization in the microenvironment in a single readout (159). It is an important clue for studies of T cells’ development and migration biology in the context of transplantation. the T cell behavior and development and cell interaction networks will be uncovered to allow for the comprehensive understanding of the rejection and tolerance mechanism through multi-omic profiling technique.

However, several limitations exist in multi-omic profiling. The first is the imperfect analysis for each omics data due to noise, and especially, drop-out accompanied by single-cell measurements, which may lead to the lack of information about mutation, alteration, or subtle expression. The second is the concomitant loss of detail within each cell due to the development of methods by incorporating more than thousands of cells. With newer and newer omics measurements such as proteomic and metabolic sequencing being incorporated (160), details of each cell are lost. Therefore, it is imperative to refine these large number of already existing multi-omics methods to obtain higher resolution and accurately measure base-level events in the genome. The ongoing emergence of multi-omics profiling approaches enables the in-depth understanding of T cells in SOT.

## Author contributions

YZ wrote the manuscript and designed the figures. GL and ML edited and revised the manuscript. All authors contributed to the article and approved the submitted version.

## References

[1] Wang P Jiang Z Wang C Liu X Li H Xu D. Immune tolerance induction using cell-based strategies in liver transplantation: Clinical perspectives. Front Immunol (2020) 11:1723. doi: 10.3389/fimmu.2020.01723 33013824 PMC7461870

[2] George PM Patterson CM Reed AK Thillai M. Lung transplantation for idiopathic pulmonary fibrosis. Lancet Respir Med (2019) 7(3):271–82. doi: 10.1016/S2213-2600(18)30502-2 30738856

[3] Guglin M Zucker MJ Borlaug BA Breen E Cleveland J Johnson MR. Evaluation for heart transplantation and LVAD implantation: JACC council perspectives. J Am Coll Cardiol (2020) 75(12):1471–87. doi: 10.1016/j.jacc.2020.01.034 32216916

[4] Courbage S Canioni D Talbotec C Lambe C Chardot C Rabant M. Beyond 10 years, with or without an intestinal graft: Present and future? Am J Transplant (2020) 20(10):2802–12. doi: 10.1111/ajt.15899 32277553

[5] Alfaro R Martinez-Banaclocha H Llorente S Jimenez-Coll V Galian JA Botella C. Computational prediction of biomarkers, pathways, and new target drugs in the pathogenesis of immune-based diseases regarding kidney transplantation rejection. Front Immunol (2021) 12:800968. doi: 10.3389/fimmu.2021.800968 34975915 PMC8714745

[6] Kroger N. Preventing graft-Versus-Host disease without losing graft-Versus-Leukemia effect after allogeneic stem-cell transplantation. J Clin Oncol (2020) 38(29):3357–60. doi: 10.1200/JCO.20.01756 32706637

[7] Marino J Paster J Benichou G. Allorecognition by T lymphocytes and allograft rejection. Front Immunol (2016) 7:582. doi: 10.3389/fimmu.2016.00582 28018349 PMC5155009

[8] Wood KJ Goto R. Mechanisms of rejection: current perspectives. Transplantation (2012) 93(1):1–10. doi: 10.1097/TP.0b013e31823cab44 22138818

[9] Hara M Kingsley CI Niimi M Read S Turvey SE Bushell AR. IL-10 is required for regulatory T cells to mediate tolerance to alloantigens in vivo. J Immunol (2001) 166(6):3789–96. doi: 10.4049/jimmunol.166.6.3789 11238621

[10] Boix F Trujillo C Muro M. Cell-mediated immunity (CMI) as the instrument to assess the response against the allograft: Present and future. Curr Protein Pept Sci (2018) 19(11):1092–106. doi: 10.2174/1389203719666180730164542 30062963

[11] Giladi A Amit I. Immunology, one cell at a time. Nature (2017) 547(7661):27–9. doi: 10.1038/547027a 28682352

[12] Linnarsson S Teichmann SA. Single-cell genomics: coming of age. Genome Biol (2016) 17:97. doi: 10.1186/s13059-016-0960-x 27160975 PMC4862185

[13] Villani AC Satija R Reynolds G Sarkizova S Shekhar K Fletcher J. Single-cell RNA-seq reveals new types of human blood dendritic cells, monocytes, and progenitors. Science (2017) 356(6335):eaah4573. doi: 10.1126/science.aah4573 28428369 PMC5775029

[14] Trapnell C Cacchiarelli D Grimsby J Pokharel P Li S Morse M. The dynamics and regulators of cell fate decisions are revealed by pseudotemporal ordering of single cells. Nat Biotechnol (2014) 32(4):381–6. doi: 10.1038/nbt.2859 PMC4122333 24658644

[15] Paul F Arkin Y Giladi A Jaitin DA Kenigsberg E Keren-Shaul H. Transcriptional heterogeneity and lineage commitment in myeloid progenitors. Cell (2015) 163(7):1663–77. doi: 10.1016/j.cell.2015.11.013 26627738

[16] Chu LF Leng N Zhang J Hou Z Mamott D Vereide DT. Single-cell RNA-seq reveals novel regulators of human embryonic stem cell differentiation to definitive endoderm. Genome Biol (2016) 17(1):173. doi: 10.1186/s13059-016-1033-x 27534536 PMC4989499

[17] Tirosh I Venteicher AS Hebert C Escalante LE Patel AP Yizhak K. Single-cell RNA-seq supports a developmental hierarchy in human oligodendroglioma. Nature (2016) 539(7628):309–13. doi: 10.1038/nature20123 PMC5465819 27806376

[18] Qiu X Mao Q Tang Y Wang L Chawla R Pliner HA. Reversed graph embedding resolves complex single-cell trajectories. Nat Methods (2017) 14(10):979–82. doi: 10.1038/nmeth.4402 PMC5764547 28825705

[19] Zhang Y Joe G Hexner E Zhu J Emerson SG. Host-reactive CD8+ memory stem cells in graft-versus-host disease. Nat Med (2005) 11(12):1299–305. doi: 10.1038/nm1326 16288282

[20] Liu Z Fan H Jiang S. CD4(+) T-cell subsets in transplantation. Immunol Rev (2013) 252(1):183–91. doi: 10.1111/imr.12038 23405905

[21] Jiang S Herrera O Lechler RI. New spectrum of allorecognition pathways: implications for graft rejection and transplantation tolerance. Curr Opin Immunol (2004) 16(5):550–7. doi: 10.1016/j.coi.2004.07.011 15341998

[22] Chan SY DeBruyne LA Goodman RE Eichwald EJ Bishop DK. *In vivo* depletion of CD8+ T cells results in Th2 cytokine production and alternate mechanisms of allograft rejection. Transplantation (1995) 59(8):1155–61. 7732563

[23] Zelenika D Adams E Mellor A Simpson E Chandler P Stockinger B. Rejection of h-y disparate skin grafts by monospecific CD4+ Th1 and Th2 cells: no requirement for CD8+ T cells or b cells. J Immunol (1998) 161(4):1868–74. 9712055

[24] Barbara JA Turvey SE Kingsley CI Spriewald BM Hara M Witzke O. Islet allograft rejection can be mediated by CD4+, alloantigen experienced, direct pathway T cells of TH1 and TH2 cytokine phenotype. Transplantation (2000) 70(11):1641–9. doi: 10.1097/00007890-200012150-00017 11152227

[25] Laan M Cui ZH Hoshino H Lotvall J Sjostrand M Gruenert DC. Neutrophil recruitment by human IL-17 *via* c-X-C chemokine release in the airways. J Immunol (1999) 162(4):2347–52. 9973514

[26] Healy DG Watson RW O'Keane C Egan JJ McCarthy JF Hurley J. Neutrophil transendothelial migration potential predicts rejection severity in human cardiac transplantation. Eur J Cardiothorac Surg (2006) 29(5):760–6. doi: 10.1016/j.ejcts.2006.01.065 16616855

[27] Rao DA Eid RE Qin L Yi T Kirkiles-Smith NC Tellides G. Interleukin (IL)-1 promotes allogeneic T cell intimal infiltration and IL-17 production in a model of human artery rejection. J Exp Med (2008) 205(13):3145–58. doi: 10.1084/jem.20081661 PMC2605225 19075290

[28] Szabo PA Levitin HM Miron M Snyder ME Senda T Yuan J. Single-cell transcriptomics of human T cells reveals tissue and activation signatures in health and disease. Nat Commun (2019) 10(1):4706. doi: 10.1038/s41467-019-12464-3 31624246 PMC6797728

[29] Hori S Nomura T Sakaguchi S. Control of regulatory T cell development by the transcription factor Foxp3. Science (2003) 299(5609):1057–61. doi: 10.1126/science.1079490 12522256

[30] Pigliucci M Murren CJ Schlichting CD. Phenotypic plasticity and evolution by genetic assimilation. J Exp Biol (2006) 209(Pt 12):2362–7. doi: 10.1242/jeb.02070 16731812

[31] Pace L. Temporal and epigenetic control of plasticity and fate decision during CD8(+) T-cell memory differentiation. Cold Spring Harb Perspect Biol (2021) 13(12):a037754. doi: 10.1101/cshperspect.a037754 PMC8635004 33972365

[32] Jameson SC Masopust D. Understanding subset diversity in T cell memory. Immunity (2018) 48(2):214–26. doi: 10.1016/j.immuni.2018.02.010 PMC5863745 29466754

[33] Nadazdin O Boskovic S Murakami T Tocco G Smith RN Colvin RB. Host alloreactive memory T cells influence tolerance to kidney allografts in nonhuman primates. Sci Transl Med (2011) 3(86):86ra51. doi: 10.1126/scitranslmed.3002093 PMC3261229 21653831

[34] Kim H Kim H Lee SK Jin XL Kim TJ Park C. Memory T cells are significantly increased in rejected liver allografts of rhesus monkeys. Liver Transpl (2018) 24(2):256–68. doi: 10.1002/lt.24983 PMC5817407 29150986

[35] Zuber J Shonts B Lau SP Obradovic A Fu J Yang S. Bidirectional intragraft alloreactivity drives the repopulation of human intestinal allografts and correlates with clinical outcome. Sci Immunol (2016) 1(4):eaah3732. doi: 10.1126/sciimmunol.aah3732 28239678 PMC5323244

[36] Saravia J Chapman NM Chi H. Helper T cell differentiation. Cell Mol Immunol (2019) 16(7):634–43. doi: 10.1038/s41423-019-0220-6 PMC6804569 30867582

[37] Luckheeram RV Zhou R Verma AD Xia B. CD4(+)T cells: differentiation and functions. Clin Dev Immunol (2012) 2012:925135. doi: 10.1155/2012/925135 22474485 PMC3312336

[38] Zhou L Chong MM Littman DR. Plasticity of CD4+ T cell lineage differentiation. Immunity (2009) 30(5):646–55. doi: 10.1016/j.immuni.2009.05.001 19464987

[39] Clambey ET Davenport B Kappler JW Marrack P Homann D. Molecules in medicine mini review: the alphabeta T cell receptor. J Mol Med (Berl) (2014) 92(7):735–41. doi: 10.1007/s00109-014-1145-2 PMC4269364 24848996

[40] Mora T. WAM. How many different clonotypes do immune repertoires contain? Curr Opin Syst Biol (2019) 18:104–10. doi: 10.1016/j.coisb.2019.10.001

[41] Davis MM Bjorkman PJ. T-Cell antigen receptor genes and T-cell recognition. Nature (1988) 334(6181):395–402. doi: 10.1038/334395a0 3043226

[42] Nikolich-Zugich J Slifka MK Messaoudi I. The many important facets of T-cell repertoire diversity. Nat Rev Immunol (2004) 4(2):123–32. doi: 10.1038/nri1292 15040585

[43] Bradley P Thomas PG. Using T cell receptor repertoires to understand the principles of adaptive immune recognition. Annu Rev Immunol (2019) 37:547–70. doi: 10.1146/annurev-immunol-042718-041757 30699000

[44] Davis MM Boyd SD. Recent progress in the analysis of alphabetaT cell and b cell receptor repertoires. Curr Opin Immunol (2019) 59:109–14. doi: 10.1016/j.coi.2019.05.012 PMC7075470 31326777

[45] Song Y Zhu Y Hu B Liu Y Lin D Jin Z. Donor gammadeltaT cells promote GVL effect and mitigate aGVHD in allogeneic hematopoietic stem cell transplantation. Front Immunol (2020) 11:558143. doi: 10.3389/fimmu.2020.558143 33178187 PMC7596318

[46] Qi Q Liu Y Cheng Y Glanville J Zhang D Lee JY. Diversity and clonal selection in the human T-cell repertoire. Proc Natl Acad Sci U.S.A. (2014) 111(36):13139–44. doi: 10.1073/pnas.1409155111 PMC4246948 25157137

[47] Pai JA Satpathy AT. High-throughput and single-cell T cell receptor sequencing technologies. Nat Methods (2021) 18(8):881–92. doi: 10.1038/s41592-021-01201-8 PMC9345561 34282327

[48] Turner SJ Doherty PC McCluskey J Rossjohn J. Structural determinants of T-cell receptor bias in immunity. Nat Rev Immunol (2006) 6(12):883–94. doi: 10.1038/nri1977 17110956

[49] Taylor JJ Pape KA Steach HR Jenkins MK. Humoral immunity. apoptosis and antigen affinity limit effector cell differentiation of a single naive b cell. Science (2015) 347(6223):784–7. doi: 10.1126/science.aaa1342 PMC4412594 25636798

[50] Tubo NJ Pagan AJ Taylor JJ Nelson RW Linehan JL Ertelt JM. Single naive CD4+ T cells from a diverse repertoire produce different effector cell types during infection. Cell (2013) 153(4):785–96. doi: 10.1016/j.cell.2013.04.007 PMC3766899 23663778

[51] DeWolf S Grinshpun B Savage T Lau SP Obradovic A Shonts B. Quantifying size and diversity of the human T cell alloresponse. JCI Insight (2018) 3(15):e121256. doi: 10.1172/jci.insight.121256 PMC6129121 30089728

[52] Suchin EJ Langmuir PB Palmer E Sayegh MH Wells AD Turka LA. Quantifying the frequency of alloreactive T cells *in vivo*: new answers to an old question. J Immunol (2001) 166(2):973–81. doi: 10.4049/jimmunol.166.2.973 11145675

[53] Dangi A Yu S Luo X. Emerging approaches and technologies in transplantation: the potential game changers. Cell Mol Immunol (2019) 16(4):334–42. doi: 10.1038/s41423-019-0207-3 PMC6462010 30760918

[54] Singh M Al-Eryani G Carswell S Ferguson JM Blackburn J Barton K. High-throughput targeted long-read single cell sequencing reveals the clonal and transcriptional landscape of lymphocytes. Nat Commun (2019) 10(1):3120. doi: 10.1038/s41467-019-11049-4 31311926 PMC6635368

[55] Perkel JM. Single-cell analysis enters the multiomics age. Nature (2021) 595(7868):614–6. doi: 10.1038/d41586-021-01994-w

[56] Hao Y Hao S Andersen-Nissen E Mauck WM Zheng S Butler A. Satija: Integrated analysis of multimodal single-cell data. Cell (2021) 184(13):3573–87.e29. doi: 10.1016/j.cell.2021.04.048 34062119 PMC8238499

[57] Engel JA Lee HJ Williams CG Kuns R Olver S Lansink LI. Single-cell transcriptomics of alloreactive CD4+ T cells over time reveals divergent fates during gut graft-versus-host disease. JCI Insight (2020) 5(13):e137990. doi: 10.1172/jci.insight.137990 32484791 PMC7406307

[58] Zemmour D Zilionis R Kiner E Klein AM Mathis D Benoist C. Single-cell gene expression reveals a landscape of regulatory T cell phenotypes shaped by the TCR. Nat Immunol (2018) 19(3):291–301. doi: 10.1038/s41590-018-0051-0 29434354 PMC6069633

[59] Ronca V Wootton G Milani C Cain O. The immunological basis of liver allograft rejection. Front Immunol (2020) 11:2155. doi: 10.3389/fimmu.2020.02155 32983177 PMC7492390

[60] Liu Y Hu J Liu D Zhou S Liao J Liao G. Single-cell analysis reveals immune landscape in kidneys of patients with chronic transplant rejection. Theranostics (2020) 10(19):8851–62. doi: 10.7150/thno.48201 PMC7392010 32754283

[61] See P Lum J Chen J Ginhoux F. A single-cell sequencing guide for immunologists. Front Immunol (2018) 9:2425. doi: 10.3389/fimmu.2018.02425 30405621 PMC6205970

[62] Babel N Stervbo U Reinke P Volk HD. The identity card of T cells-clinical utility of T-cell receptor repertoire analysis in transplantation. Transplantation (2019) 103(8):1544–55. doi: 10.1097/TP.0000000000002776 31033649

[63] Chen H Ye F Guo G. Revolutionizing immunology with single-cell RNA sequencing. Cell Mol Immunol (2019) 16(3):242–9. doi: 10.1038/s41423-019-0214-4 PMC6460502 30796351

[64] Scandling JD Busque S Dejbakhsh-Jones S Benike C Millan MT Shizuru JA. Tolerance and chimerism after renal and hematopoietic-cell transplantation. N Engl J Med (2008) 358(4):362–8. doi: 10.1056/NEJMoa074191 18216356

[65] Zuber J Sykes M. Mechanisms of mixed chimerism-based transplant tolerance. Trends Immunol (2017) 38(11):829–43. doi: 10.1016/j.it.2017.07.008 PMC5669809 28826941

[66] Kawai T Leventhal J Wood K Strober S. Summary of the third international workshop on clinical tolerance. Am J Transplant (2019) 19(2):324–30. doi: 10.1111/ajt.15086 PMC6349553 30133954

[67] Malone AF Wu H Fronick C Fulton R Gaut JP Humphreys BD. Harnessing expressed single nucleotide variation and single cell RNA sequencing to define immune cell chimerism in the rejecting kidney transplant. J Am Soc Nephrol (2020) 31(9):1977–86. doi: 10.1681/ASN.2020030326 PMC7461682 32669324

[68] Jiang Y Ding Q Xie X Libby RT Lefebvre V Gan L. Transcription factors SOX4 and SOX11 function redundantly to regulate the development of mouse retinal ganglion cells. J Biol Chem (2013) 288(25):18429–38. doi: 10.1074/jbc.M113.478503 PMC3689985 23649630

[69] Gadi J Jung SH Lee MJ Jami A Ruthala K Kim KM. The transcription factor protein Sox11 enhances early osteoblast differentiation by facilitating proliferation and the survival of mesenchymal and osteoblast progenitors. J Biol Chem (2013) 288(35):25400–13. doi: 10.1074/jbc.M112.413377 PMC3757203 23888050

[70] Snyder ME Finlayson MO Connors TJ Dogra P Senda T Bush E. Generation and persistence of human tissue-resident memory T cells in lung transplantation. Sci Immunol (2019) 4(33):eaav5581. doi: 10.1126/sciimmunol.aav5581 30850393 PMC6435356

[71] Fu J Zuber J Shonts B Obradovic A Wang Z Frangaj K. Lymphohematopoietic graft-versus-host responses promote mixed chimerism in patients receiving intestinal transplantation. J Clin Invest (2021) 131(8):e141698. doi: 10.1172/JCI141698 33630757 PMC8062082

[72] Melvin AJ McGurn ME Bort SJ Gibson C Lewis DB. Hypomethylation of the interferon-gamma gene correlates with its expression by primary T-lineage cells. Eur J Immunol (1995) 25(2):426–30. doi: 10.1002/eji.1830250218 7875204

[73] Komori HK Hart T LaMere SA Chew PV Salomon DR. Defining CD4 T cell memory by the epigenetic landscape of CpG DNA methylation. J Immunol (2015) 194(4):1565–79. doi: 10.4049/jimmunol.1401162 PMC4364524 25576597

[74] Smallwood SA Lee HJ Angermueller C Krueger F Saadeh H Peat J. Single-cell genome-wide bisulfite sequencing for assessing epigenetic heterogeneity. Nat Methods (2014) 11(8):817–20. doi: 10.1038/nmeth.3035 PMC4117646 25042786

[75] Guo H Zhu P Wu X Li X Wen L Tang F. Single-cell methylome landscapes of mouse embryonic stem cells and early embryos analyzed using reduced representation bisulfite sequencing. Genome Res (2013) 23(12):2126–35. doi: 10.1101/gr.161679.113 PMC3847781 24179143

[76] Macaulay IC Haerty W Kumar P Li YI Hu TX Teng MJ. G&T-seq: parallel sequencing of single-cell genomes and transcriptomes. Nat Methods (2015) 12(6):519–22. doi: 10.1038/nmeth.3370 25915121

[77] Angermueller C Clark SJ Lee HJ Macaulay IC Teng MJ Hu TX. Parallel single-cell sequencing links transcriptional and epigenetic heterogeneity. Nat Methods (2016) 13(3):229–32. doi: 10.1038/nmeth.3728 PMC4770512 26752769

[78] Yang R Cheng S Luo N Gao R Yu K Kang B. Distinct epigenetic features of tumor-reactive CD8+ T cells in colorectal cancer patients revealed by genome-wide DNA methylation analysis. Genome Biol (2019) 21(1):2. doi: 10.1186/s13059-019-1921-y 31892342 PMC6937914

[79] Buenrostro JD Giresi PG Zaba LC Chang HY Greenleaf WJ. Transposition of native chromatin for fast and sensitive epigenomic profiling of open chromatin, DNA-binding proteins and nucleosome position. Nat Methods (2013) 10(12):1213–8. doi: 10.1038/nmeth.2688 PMC3959825 24097267

[80] Buenrostro JD Wu B Litzenburger UM Ruff D Gonzales ML Snyder MP. Single-cell chromatin accessibility reveals principles of regulatory variation. Nature (2015) 523(7561):486–90. doi: 10.1038/nature14590 PMC4685948 26083756

[81] Corces MR Buenrostro JD Wu B Greenside PG Chan SM Koenig JL. Lineage-specific and single-cell chromatin accessibility charts human hematopoiesis and leukemia evolution. Nat Genet (2016) 48(10):1193–203. doi: 10.1038/ng.3646 PMC5042844 27526324

[82] Cao J Cusanovich DA Ramani V Aghamirzaie D Pliner HA Hill AJ. Joint profiling of chromatin accessibility and gene expression in thousands of single cells. Science (2018) 361(6409):1380–5. doi: 10.1126/science.aau0730 PMC6571013 30166440

[83] Liu L Liu C Quintero A Wu L Yuan Y Wang M. Deconvolution of single-cell multi-omics layers reveals regulatory heterogeneity. Nat Commun (2019) 10(1):470. doi: 10.1038/s41467-018-08205-7 30692544 PMC6349937

[84] Xing QR Farran CAE Zeng YY Yi Y Warrier T Gautam P. Parallel bimodal single-cell sequencing of transcriptome and chromatin accessibility. Genome Res (2020) 30(7):1027–39. doi: 10.1101/gr.257840.119 PMC7397874 32699019

[85] Rosenberg AB Roco CM Muscat RA Kuchina A Sample P Yao Z. Single-cell profiling of the developing mouse brain and spinal cord with split-pool barcoding. Science (2018) 360(6385):176–82. doi: 10.1126/science.aam8999 PMC7643870 29545511

[86] Ma S Zhang B LaFave LM Earl AS Chiang Z Hu Y. Chromatin potential identified by shared single-cell profiling of RNA and chromatin. Cell (2020) 183(4):1103–16.e20. doi: 10.1016/j.cell.2020.09.056 33098772 PMC7669735

[87] Macosko EZ Basu A Satija R Nemesh J Shekhar K Goldman M. Highly parallel genome-wide expression profiling of individual cells using nanoliter droplets. Cell (2015) 161(5):1202–14. doi: 10.1016/j.cell.2015.05.002 PMC4481139 26000488

[88] Lu B Dong L Yi D Zhang M Zhu C Li X. Transposase-assisted tagmentation of RNA/DNA hybrid duplexes. Elife (2020) 9:e54919. doi: 10.7554/eLife.54919 32701057 PMC7402673

[89] Xu W Yang W Zhang Y Chen Y Hong N Zhang Q. ISSAAC-seq enables sensitive and flexible multimodal profiling of chromatin accessibility and gene expression in single cells. Nat Methods (2022) 19(10):1243–9. doi: 10.1038/s41592-022-01601-4 36109677

[90] Hogquist KA Jameson SC. The self-obsession of T cells: how TCR signaling thresholds affect fate 'decisions' and effector function. Nat Immunol (2014) 15(9):815–23. doi: 10.1038/ni.2938 PMC4348363 25137456

[91] Stritesky GL Jameson SC Hogquist KA. Selection of self-reactive T cells in the thymus. Annu Rev Immunol (2012) 30:95–114. doi: 10.1146/annurev-immunol-020711-075035 22149933 PMC3518413

[92] Taniuchi I. CD4 helper and CD8 cytotoxic T cell differentiation. Annu Rev Immunol (2018) 36:579–601. doi: 10.1146/annurev-immunol-042617-053411 29677476

[93] Xiong Y Bosselut R. CD4-CD8 differentiation in the thymus: connecting circuits and building memories. Curr Opin Immunol (2012) 24(2):139–45. doi: 10.1016/j.coi.2012.02.002 PMC3773541 22387323

[94] McDonald BD Jabri B Bendelac A. Diverse developmental pathways of intestinal intraepithelial lymphocytes. Nat Rev Immunol (2018) 18(8):514–25. doi: 10.1038/s41577-018-0013-7 PMC6063796 29717233

[95] Ruscher R Hogquist KA. Development, ontogeny, and maintenance of TCRalphabeta(+) CD8alphaalpha IEL. Curr Opin Immunol (2019) 58:83–8. doi: 10.1016/j.coi.2019.04.010 PMC6612447 31146182

[96] Li MO Rudensky AY. T Cell receptor signalling in the control of regulatory T cell differentiation and function. Nat Rev Immunol (2016) 16(4):220–33. doi: 10.1038/nri.2016.26 PMC4968889 27026074

[97] Yoshida H Lareau CA Ramirez RN Rose SA Maier B Wroblewska A. The cis-regulatory atlas of the mouse immune system. Cell (2019) 176(4):897–912.e20. doi: 10.1016/j.cell.2018.12.036 30686579 PMC6785993

[98] Mingueneau M Kreslavsky T Gray D Heng T Cruse R Ericson J. The transcriptional landscape of alphabeta T cell differentiation. Nat Immunol (2013) 14(6):619–32. doi: 10.1038/ni.2590 PMC3660436 23644507

[99] Chopp LB Gopalan V Ciucci T Ruchinskas A Rae Z Lagarde M. An integrated epigenomic and transcriptomic map of mouse and human alphabeta T cell development. Immunity (2020) 53(6):1182–201.e8. doi: 10.1016/j.immuni.2020.10.024 33242395 PMC8641659

[100] Peterson VM Zhang KX Kumar N Wong J Li L Wilson DC. Multiplexed quantification of proteins and transcripts in single cells. Nat Biotechnol (2017) 35(10):936–9. doi: 10.1038/nbt.3973 28854175

[101] Fredriksson S Gullberg M Jarvius J Olsson C Pietras K Gustafsdottir SM. Protein detection using proximity-dependent DNA ligation assays. Nat Biotechnol (2002) 20(5):473–7. doi: 10.1038/nbt0502-473 11981560

[102] Darmanis S Gallant CJ Marinescu VD Niklasson M Segerman A Flamourakis G. Simultaneous multiplexed measurement of RNA and proteins in single cells. Cell Rep (2016) 14(2):380–9. doi: 10.1016/j.celrep.2015.12.021 PMC4713867 26748716

[103] Genshaft AS Li S Gallant CJ Darmanis S Prakadan SM Ziegler CG. Multiplexed, targeted profiling of single-cell proteomes and transcriptomes in a single reaction. Genome Biol (2016) 17(1):188. doi: 10.1186/s13059-016-1045-6 27640647 PMC5027636

[104] Stahlberg A Thomsen C Ruff D Aman P. Quantitative PCR analysis of DNA, RNAs, and proteins in the same single cell. Clin Chem (2012) 58(12):1682–91. doi: 10.1373/clinchem.2012.191445 23014600

[105] Frei AP Bava FA Zunder ER Hsieh EW Chen SY Nolan GP. Highly multiplexed simultaneous detection of RNAs and proteins in single cells. Nat Methods (2016) 13(3):269–75. doi: 10.1038/nmeth.3742 PMC4767631 26808670

[106] Ogbeide S Giannese F Mincarelli L Macaulay IC. Into the multiverse: advances in single-cell multiomic profiling. Trends Genet (2022) 38(8):831–43. doi: 10.1016/j.tig.2022.03.015 35537880

[107] Reimegard J Tarbier M Danielsson M Schuster J Baskaran S Panagiotou S. A combined approach for single-cell mRNA and intracellular protein expression analysis. Commun Biol (2021) 4(1):624. doi: 10.1038/s42003-021-02142-w 34035432 PMC8149646

[108] Stoeckius M Hafemeister C Stephenson W Houck-Loomis B Chattopadhyay PK Swerdlow H. Simultaneous epitope and transcriptome measurement in single cells. Nat Methods (2017) 14(9):865–8. doi: 10.1038/nmeth.4380 PMC5669064 28759029

[109] Budnik B Levy E Harmange G Slavov N. SCoPE-MS: mass spectrometry of single mammalian cells quantifies proteome heterogeneity during cell differentiation. Genome Biol (2018) 19(1):161. doi: 10.1186/s13059-018-1547-5 30343672 PMC6196420

[110] Specht H Emmott E Petelski AA Huffman RG Perlman DH Serra M. Single-cell proteomic and transcriptomic analysis of macrophage heterogeneity using SCoPE2. Genome Biol (2021) 22(1):50. doi: 10.1186/s13059-021-02267-5 33504367 PMC7839219

[111] Fiskin E Lareau CA Ludwig LS Eraslan G Liu F Ring AM. Single-cell profiling of proteins and chromatin accessibility using PHAGE-ATAC. Nat Biotechnol (2022) 40(3):374–81. doi: 10.1038/s41587-021-01065-5 PMC10549956 34675424

[112] Macedo C Orkis EA Popescu I Elinoff BD Zeevi A Shapiro R. Contribution of naive and memory T-cell populations to the human alloimmune response. Am J Transplant (2009) 9(9):2057–66. doi: 10.1111/j.1600-6143.2009.02742.x 19624567

[113] Golshayan D Wyss JC Buckland M Hernandez-Fuentes M Lechler RI. Differential role of naive and memory CD4 T-cell subsets in primary alloresponses. Am J Transplant (2010) 10(8):1749–59. doi: 10.1111/j.1600-6143.2010.03180.x 20659087

[114] Oakes T Heather JM Best K Byng-Maddick R Husovsky C Ismail M. Quantitative characterization of the T cell receptor repertoire of naive and memory subsets using an integrated experimental and computational pipeline which is robust, economical, and versatile. Front Immunol (2017) 8:1267. doi: 10.3389/fimmu.2017.01267 29075258 PMC5643411

[115] Morris H DeWolf S Robins H Sprangers B LoCascio SA Shonts BA. Tracking donor-reactive T cells: Evidence for clonal deletion in tolerant kidney transplant patients. Sci Transl Med (2015) 7(272):272ra10. doi: 10.1126/scitranslmed.3010760 PMC4360892 25632034

[116] Obradovic A Shen Y Sykes M Fu J. Integrated analysis toolset for defining and tracking alloreactive T-cell clones after human solid organ and hematopoietic stem cell transplantation. Softw Impacts (2021) 10:100142. doi: 10.1016/j.simpa.2021.100142 PMC8920412 35291378

[117] Aschauer C Jelencsics K Hu K Heinzel A Vetter J Fraunhofer T. Next generation sequencing based assessment of the alloreactive T cell receptor repertoire in kidney transplant patients during rejection: a prospective cohort study. BMC Nephrol (2019) 20(1):346. doi: 10.1186/s12882-019-1541-5 31477052 PMC6719356

[118] Savage TM Shonts BA Lau S Obradovic A Robins H Shaked A. Deletion of donor-reactive T cell clones after human liver transplant. Am J Transplant (2020) 20(2):538–45. doi: 10.1111/ajt.15592 PMC6984984 31509321

[119] Li M Song S Tian G Zhi Y Chen Y Huang H. Expansion kinetics of graft-versus-host T cell clones in patients with post-liver transplant graft-versus-host disease. Am J Transplant (2022) 22(11):2689–93. doi: 10.1111/ajt.17112 35665999

[120] Tian G Li M Lv G. Analysis of T-cell receptor repertoire in transplantation: Fingerprint of T cell-mediated alloresponse. Front Immunol (2021) 12:778559. doi: 10.3389/fimmu.2021.778559 35095851 PMC8790170

[121] Thome JJ Grinshpun B Kumar BV Kubota M Ohmura Y Lerner H. Longterm maintenance of human naive T cells through *in situ* homeostasis in lymphoid tissue sites. Sci Immunol (2016) 1(6):eaah6506. doi: 10.1126/sciimmunol.aah6506 28361127 PMC5367636

[122] Fu J Zuber J Martinez M Shonts B Obradovic A Wang H. Human intestinal allografts contain functional hematopoietic stem and progenitor cells that are maintained by a circulating pool. Cell Stem Cell (2019) 24(2):227–39.e8. doi: 10.1016/j.stem.2018.11.007 30503142 PMC6398344

[123] Taubert R Pischke S Schlue J Wedemeyer H Noyan F Heim A. Enrichment of regulatory T cells in acutely rejected human liver allografts. Am J Transplant (2012) 12(12):3425–36. doi: 10.1111/j.1600-6143.2012.04264.x 22994589

[124] Taubert R Hardtke-Wolenski M Noyan F Wilms A Baumann AK Schlue J. Intrahepatic regulatory T cells in autoimmune hepatitis are associated with treatment response and depleted with current therapies. J Hepatol (2014) 61(5):1106–14. doi: 10.1016/j.jhep.2014.05.034 24882050

[125] Huibers MM Gareau AJ Vink A Kruit R Feringa H Beerthuijzen JM. The composition of ectopic lymphoid structures suggests involvement of a local immune response in cardiac allograft vasculopathy. J Heart Lung Transplant (2015) 34(5):734–45. doi: 10.1016/j.healun.2014.11.022 25655346

[126] Mederacke YS Nienen M Jarek M Geffers R Hupa-Breier K Babel N. T Cell receptor repertoires within liver allografts are different to those in the peripheral blood. J Hepatol (2021) 74(5):1167–75. doi: 10.1016/j.jhep.2020.12.014 33347951

[127] Yang G Ou M Chen H Guo C Chen J Lin H. Characteristic analysis of TCR beta-chain CDR3 repertoire for pre- and post-liver transplantation. Oncotarget (2018) 9(77):34506–19. doi: 10.18632/oncotarget.26138 PMC6195376 30349645

[128] Han FF Fan H Ren LL Wang HG Wang C Ma X. Profiling the pattern of human TRB/IGH-CDR3 repertoire in liver transplantation patients *via* high-throughput sequencing analysis. Scand J Immunol (2020) 92(2):e12912. doi: 10.1111/sji.12912 32458431

[129] Habal MV Miller AMI Rao S Lin S Obradovic A Khosravi-Maharlooei M. T Cell repertoire analysis suggests a prominent bystander response in human cardiac allograft vasculopathy. Am J Transplant (2021) 21(4):1465–76. doi: 10.1111/ajt.16333 PMC8672660 33021057

[130] Satpathy AT Saligrama N Buenrostro JD Wei Y Wu B Rubin AJ. Transcript-indexed ATAC-seq for precision immune profiling. Nat Med (2018) 24(5):580–90. doi: 10.1038/s41591-018-0008-8 PMC5948148 29686426

[131] Birnbaum ME Mendoza JL Sethi DK Dong S Glanville J Dobbins J. Deconstructing the peptide-MHC specificity of T cell recognition. Cell (2014) 157(5):1073–87. doi: 10.1016/j.cell.2014.03.047 PMC4071348 24855945

[132] Newell EW Davis MM. Beyond model antigens: high-dimensional methods for the analysis of antigen-specific T cells. Nat Biotechnol (2014) 32(2):149–57. doi: 10.1038/nbt.2783 PMC4001742 24441473

[133] Svensson V Natarajan KN Ly LH Miragaia RJ Labalette C Macaulay IC. Power analysis of single-cell RNA-sequencing experiments. Nat Methods (2017) 14(4):381–7. doi: 10.1038/nmeth.4220 PMC5376499 28263961

[134] Manzoni C Kia DA Vandrovcova J Hardy J Wood NW Lewis PA. Genome, transcriptome and proteome: the rise of omics data and their integration in biomedical sciences. Brief Bioinform (2018) 19(2):286–302. doi: 10.1093/bib/bbw114 27881428 PMC6018996

[135] Mimitou EP Lareau CA Chen KY Zorzetto-Fernandes AL Hao Y Takeshima Y. Scalable, multimodal profiling of chromatin accessibility, gene expression and protein levels in single cells. Nat Biotechnol (2021) 39(10):1246–58. doi: 10.1038/s41587-021-00927-2 PMC8763625 34083792

[136] Swanson E Lord C Reading J Heubeck AT Genge PC Thomson Z. Simultaneous trimodal single-cell measurement of transcripts, epitopes, and chromatin accessibility using TEA-seq. Elife (2021) 10:e63632 doi: 10.7554/eLife.63632 33835024 PMC8034981

[137] Mirza B Wang W Wang J Choi H Chung NC Ping P. Machine learning and integrative analysis of biomedical big data. Genes (Basel) (2019) 10(2):87. doi: 10.3390/genes10020087 PMC6410075 30696086

[138] Saviano A Henderson NC Baumert TF. Single-cell genomics and spatial transcriptomics: Discovery of novel cell states and cellular interactions in liver physiology and disease biology. J Hepatol (2020) 73(5):1219–30. doi: 10.1016/j.jhep.2020.06.004 PMC7116221 32534107

[139] Hu KH Eichorst JP McGinnis CS Patterson DM Chow ED Kersten K. ZipSeq: barcoding for real-time mapping of single cell transcriptomes. Nat Methods (2020) 17(8):833–43. doi: 10.1038/s41592-020-0880-2 PMC7891292 32632238

[140] Stahl PL Salmen F Vickovic S Lundmark A Navarro JF Magnusson J. Visualization and analysis of gene expression in tissue sections by spatial transcriptomics. Science (2016) 353(6294):78–82. doi: 10.1126/science.aaf2403 27365449

[141] Rodriques SG Stickels RR Goeva A Martin CA Murray E Vanderburg CR. Slide-seq: A scalable technology for measuring genome-wide expression at high spatial resolution. Science (2019) 363(6434):1463–7. doi: 10.1126/science.aaw1219 PMC6927209 30923225

[142] Vickovic S Eraslan G Salmen F Klughammer J Stenbeck L Schapiro D. High-definition spatial transcriptomics for *in situ* tissue profiling. Nat Methods (2019) 16(10):987–90. doi: 10.1038/s41592-019-0548-y PMC6765407 31501547

[143] Korsunsky I Millard N Fan J Slowikowski K Zhang F Wei K. Fast, sensitive and accurate integration of single-cell data with harmony. Nat Methods (2019) 16(12):1289–96. doi: 10.1038/s41592-019-0619-0 PMC6884693 31740819

[144] Hie B Bryson B Berger B. Efficient integration of heterogeneous single-cell transcriptomes using scanorama. Nat Biotechnol (2019) 37(6):685–91. doi: 10.1038/s41587-019-0113-3 PMC6551256 31061482

[145] Johnson WE Li C Rabinovic A. Adjusting batch effects in microarray expression data using empirical bayes methods. Biostatistics (2007) 8(1):118–27. doi: 10.1093/biostatistics/kxj037 16632515

[146] Welch JD Kozareva V Ferreira A Vanderburg C Martin C Macosko EZ. Single-cell multi-omic integration compares and contrasts features of brain cell identity. Cell (2019) 177(7):1873–87.e17. doi: 10.1016/j.cell.2019.05.006 31178122 PMC6716797

[147] Kharchenko PV Silberstein L Scadden DT. Bayesian Approach to single-cell differential expression analysis. Nat Methods (2014) 11(7):740–2. doi: 10.1038/nmeth.2967 PMC4112276 24836921

[148] Lin Y Ghazanfar S Wang KYX Gagnon-Bartsch JA Lo KK Su X. scMerge leverages factor analysis, stable expression, and pseudoreplication to merge multiple single-cell RNA-seq datasets. Proc Natl Acad Sci U.S.A. (2019) 116(20):9775–84. doi: 10.1073/pnas.1820006116 PMC6525515 31028141

[149] Tran HTN Ang KS Chevrier M Zhang X Lee NYS Goh M. A benchmark of batch-effect correction methods for single-cell RNA sequencing data. Genome Biol (2020) 21(1):12. doi: 10.1186/s13059-019-1850-9 31948481 PMC6964114

[150] Stuart T Butler A Hoffman P Hafemeister C Papalexi E Mauck WM. Satija: Comprehensive integration of single-cell data. Cell (2019) 177(7):1888–902.e21. doi: 10.1016/j.cell.2019.05.031 31178118 PMC6687398

[151] Singh A Shannon CP Gautier B Rohart F Vacher M Tebbutt SJ. DIABLO: an integrative approach for identifying key molecular drivers from multi-omics assays. Bioinformatics (2019) 35(17):3055–62. doi: 10.1093/bioinformatics/bty1054 PMC6735831 30657866

[152] Gasperini M Hill AJ McFaline-Figueroa JL Martin B Kim S Zhang MD. A genome-wide framework for mapping gene regulation *via* cellular genetic screens. Cell (2019) 176(1-2):377–90.e19. doi: 10.1016/j.cell.2018.11.029 30612741 PMC6690346

[153] Meng C Kuster B Culhane AC Gholami AM. A multivariate approach to the integration of multi-omics datasets. BMC Bioinf (2014) 15:162. doi: 10.1186/1471-2105-15-162 PMC4053266 24884486

[154] Lock EF Hoadley KA Marron JS Nobel AB. Joint and individual variation explained (Jive) for integrated analysis of multiple data types. Ann Appl Stat (2013) 7(1):523–42. doi: 10.1214/12-AOAS597 PMC3671601 23745156

[155] Welch JD Hartemink AJ Prins JF. MATCHER: manifold alignment reveals correspondence between single cell transcriptome and epigenome dynamics. Genome Biol (2017) 18(1):138. doi: 10.1186/s13059-017-1269-0 28738873 PMC5525279

[156] Liu J Huang Y Singh R Vert JP Noble WS. Jointly embedding multiple single-cell omics measurements. Algorithms Bioinform 143 (2019) 143:10. doi: 10.4230/LIPIcs.WABI.2019.10 PMC8496402 34632462

[157] Schep AN Wu B Buenrostro JD Greenleaf WJ. chromVAR: inferring transcription-factor-associated accessibility from single-cell epigenomic data. Nat Methods (2017) 14(10):975–8. doi: 10.1038/nmeth.4401 PMC5623146 28825706

[158] Xu C Tao D Xu C. A survey on multi-view learning (2013). Available at: https://arxiv.org/abs/1304.5634.

[159] Macaulay IC Ponting CP Voet T. Single-cell multiomics: Multiple measurements from single cells. Trends Genet (2017) 33(2):155–68. doi: 10.1016/j.tig.2016.12.003 PMC5303816 28089370

[160] Su Y Ko ME Cheng H Zhu R Xue M Wang J. Multi-omic single-cell snapshots reveal multiple independent trajectories to drug tolerance in a melanoma cell line. Nat Commun (2020) 11(1):2345. doi: 10.1038/s41467-020-15956-9 32393797 PMC7214418

